# Application of deep learning for automated diagnosis and classification of hip dysplasia on plain radiographs

**DOI:** 10.1186/s12891-024-07244-0

**Published:** 2024-02-09

**Authors:** Martin Magnéli, Alireza Borjali, Eiji Takahashi, Michael Axenhus, Henrik Malchau, Orhun K. Moratoglu, Kartik M. Varadarajan

**Affiliations:** 1 Department of Orthopaedic Surgery, Harvard Medical School, Boston, MA USA; 2 Department of Orthopaedic Surgery, Harris Orthopaedics Laboratory, Massachusetts General Hospital, Boston, MA USA; 3 Karolinska Institutet, Department of Clinical Sciences, Danderyd Hospital, Stockholm, Sweden; 4 Department of Orthopaedic Surgery, Kanazawa Medical University, Uchinada, Japan; 5 Department of Orthopaedic Surgery, Danderyd Hospital, Stockholm, Sweden; 6 Department of Orthopaedic Surgery, Sahlgrenska University Hospital, Gothenburg, Sweden

**Keywords:** Crowe classification, Deep learning model, Hartofilakidis classification, Hip dysplasia, Radiographs

## Abstract

**Background:**

Hip dysplasia is a condition where the acetabulum is too shallow to support the femoral head and is commonly considered a risk factor for hip osteoarthritis. The objective of this study was to develop a deep learning model to diagnose hip dysplasia from plain radiographs and classify dysplastic hips based on their severity.

**Methods:**

We collected pelvic radiographs of 571 patients from two single-center cohorts and one multicenter cohort. The radiographs were split in half to create hip radiographs (*n* = 1022). One orthopaedic surgeon and one resident assessed the radiographs for hip dysplasia on either side. We used the center edge (CE) angle as the primary diagnostic criteria. Hips with a CE angle < 20°, 20° to 25°, and > 25° were labeled as dysplastic, borderline, and normal, respectively. The dysplastic hips were also classified with both Crowe and Hartofilakidis classification of dysplasia. The dataset was divided into train, validation, and test subsets using 80:10:10 split-ratio that were used to train two deep learning models to classify images into normal, borderline and (1) Crowe grade 1–4 or (2) Hartofilakidis grade 1–3. A pre-trained on Imagenet VGG16 convolutional neural network (CNN) was utilized by performing layer-wise fine-turning.

**Results:**

Both models struggled with distinguishing between normal and borderline hips. However, achieved high accuracy (Model 1: 92.2% and Model 2: 83.3%) in distinguishing between normal/borderline vs. dysplastic hips. The overall accuracy of Model 1 was 68% and for Model 2 73.5%. Most misclassifications for the Crowe and Hartofilakidis classifications were +/- 1 class from the correct class.

**Conclusions:**

This pilot study shows promising results that a deep learning model distinguish between normal and dysplastic hips with high accuracy. Future research and external validation are warranted regarding the ability of deep learning models to perform complex tasks such as identifying and classifying disorders using plain radiographs.

**Level of Evidence:**

Diagnostic level IV

## Introduction

**Fig. 1:**
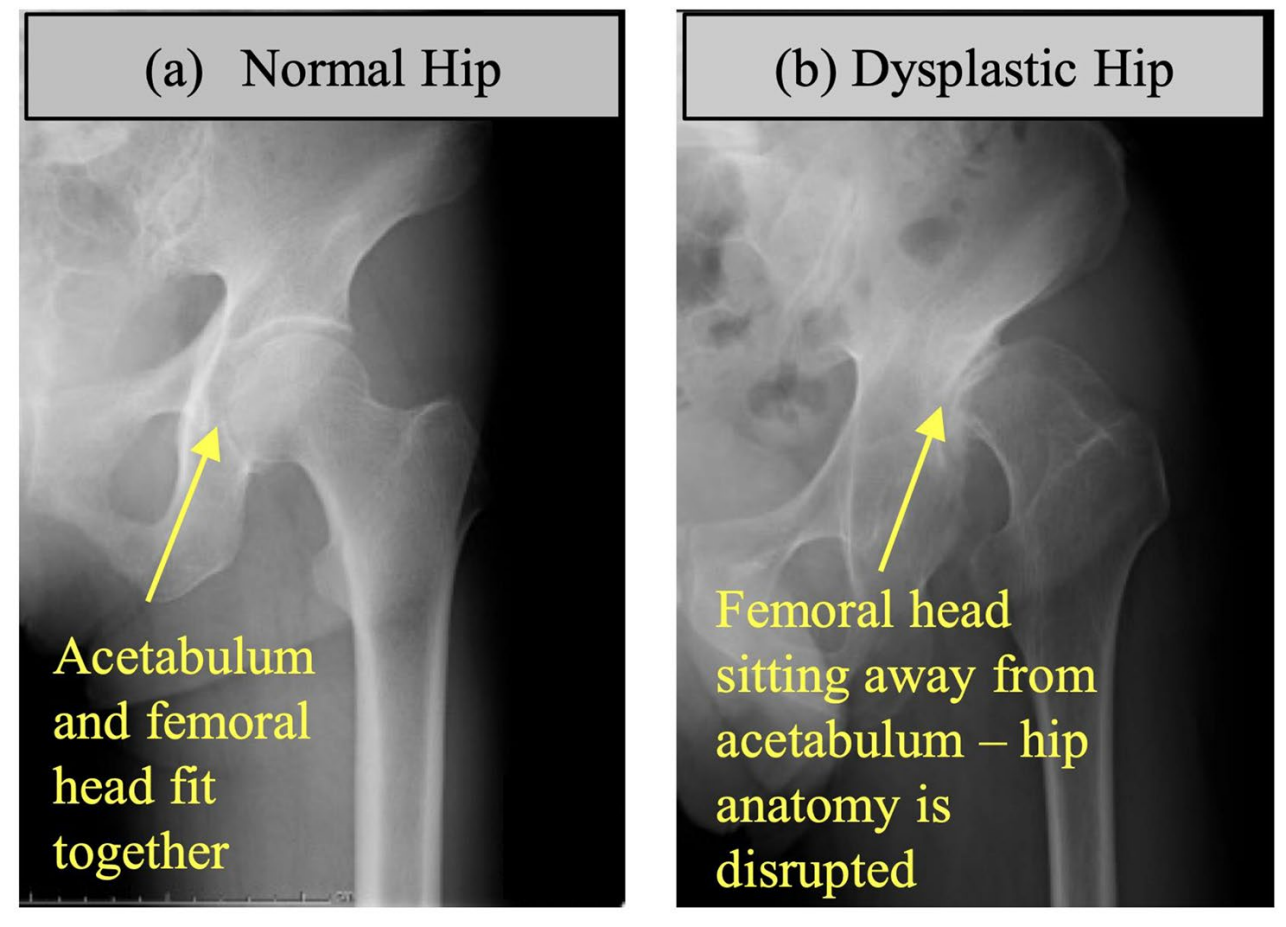
Examples of (**a**) normal, and (**b**) dysplastic hip radiograph

Hip dysplasia is a condition where the acetabulum is too shallow to support the femoral head, which can lead to subluxation or luxation of the femoral head and disrupted anatomy of the hip joint (Fig. 1). The biomechanics of a dysplastic hip joint can cause labrum tear and cartilage damage due to abnormal center of rotation and load distribution. Hip dysplasia is commonly considered a risk factor for hip osteoarthritis (OA) [1]. Surgical interventions such as periacetabular osteotomy, could affect hip OA development within this population. Hence, accurate and timely diagnosis of hip dysplasia is of utmost importance.

Congenital hip dysplasia is usually diagnosed during routine screening of newborns [2]. Hip dysplasia can also manifest later in life in adolescence. Plain radiographs of the pelvis and hip are the foundation of diagnosing adult hip dysplasia. A recent study found that among general radiologists (and thus among the reports to general practitioners), the diagnosis of most (93%) adult hip dysplasia goes unrecognized and hence untreated [3]. Furthermore, the agreement between individual orthopaedic specialists reading the same radiographs to diagnose hip dysplasia has been shown to be highly variable, with weighted kappa coefficients ranging from 0.43 to 0.93 [4–6] (from moderate to almost perfect agreement).

Moreover, the prevalence of hip dysplasia varies in different demographics from 1– 13% [7–11]. The relative rarity and demographic variation in the incidence of hip dysplasia also mean that orthopaedic practitioners are not equally familiar with the diagnosis and management of hip dysplasia. Currently, no tool is available to help orthopaedic practitioners with a better diagnosis of hip dysplasia.

Deep learning is a relatively new sub-category of artificial intelligence mainly concerned with image analysis and pattern recognition. In recent years deep learning models have been successfully used for automated image analysis and diagnosis of different orthopaedic disorders. These studies have focused on a myriad of orthopaedic disorders such as diagnosis of total hip replacement (THR) aseptic loosening, detecting the type of a THR implant prior to the revision surgery, diagnosing hard to detect tibiofemoral cartilage defects, and bone fracture detection and classification to name a few [12–19]. In most of these applications, deep learning models have achieved on-par or better performance compared to orthopaedic practitioners performing the same task. Despite these successful applications, no deep learning model has been developed so far to diagnose hip dysplasia from plain radiographs. Deep learning models may offer a solution to overcome the current challenges in the radiographic diagnosis of hip dysplasia and provide a valuable tool for reducing the inter-reader variability, enabling inexperienced practitioners to find dysplasia cases in larger cohorts, and standardizing the reporting of clinical outcomes of dysplasia patients based on disease severity. Hence, the objective of this study was to develop a deep learning model to diagnose hip dysplasia from plain radiographs and classify dysplastic hips based on their severity.

## Methods

### Study design

We conducted a retrospective study with institutional review board (IRB) approval, using previously collected imaging data. The study goal was to create and validate deep learning models for diagnosing hip dysplasia and classifying its severity from plain hip radiographs.

### Data

#### Data sources

The following three data sources were used in this study:

1. A prospective, international, multicenter study established in 2007, with the primary purpose of evaluating the outcomes of patients treated with total hip arthroplasty using vitamin-E infused highly cross-linked polyethylene liners [20]. The study consists of 16 centers in 8 countries.
2. A retrospective single center cohort study from a Japanese hospital, with the primary purpose of studying outcomes following hip arthroplasty surgery.
3. A retrospective single center cohort study from a Japanese hospital, with the primary purpose of studying dislocations following hip arthroplasty surgery.

All hospitals and the number of included hips are listed in Table 1. All patients were 18 years or older. Indication for all patients were degenerative joint disease, primary or secondary osteoarthritis. The contralateral hip was also assessed. We found no hips with Perthes disease, prior fracture or epiphysiolysis.

### Ethics

For data source 1, written informed consent was collected for all participants in the clinical study. A separate IRB approval for continuing radiographic analysis exists, Partners Human Research, protocol no. 2007P001955. For data source 2 and 3, written informed consent was collected for all participants in the clinical study. IRB committee of Kanazawa Medical University. Receipt number: 134, date of approval: June 18, 2012.

#### Data preprocessing

The images existed in both JPEG and DICOM format. DICOM images were exported into JPEG format and kept as full-size images. All patient data was removed from the DICOMs. All images were anteroposterior pelvis radiographs that were subsequently cropped by a vertical split through the symphysis, creating two AP hip images. We only included the native hips and excluded hips with arthroplasty, prior osteotomy surgery, femoral head necrosis, and scanned radiographs with overlaying free-drawn preoperative planning templates and writing.

### Ground truth

**Fig. 2:**
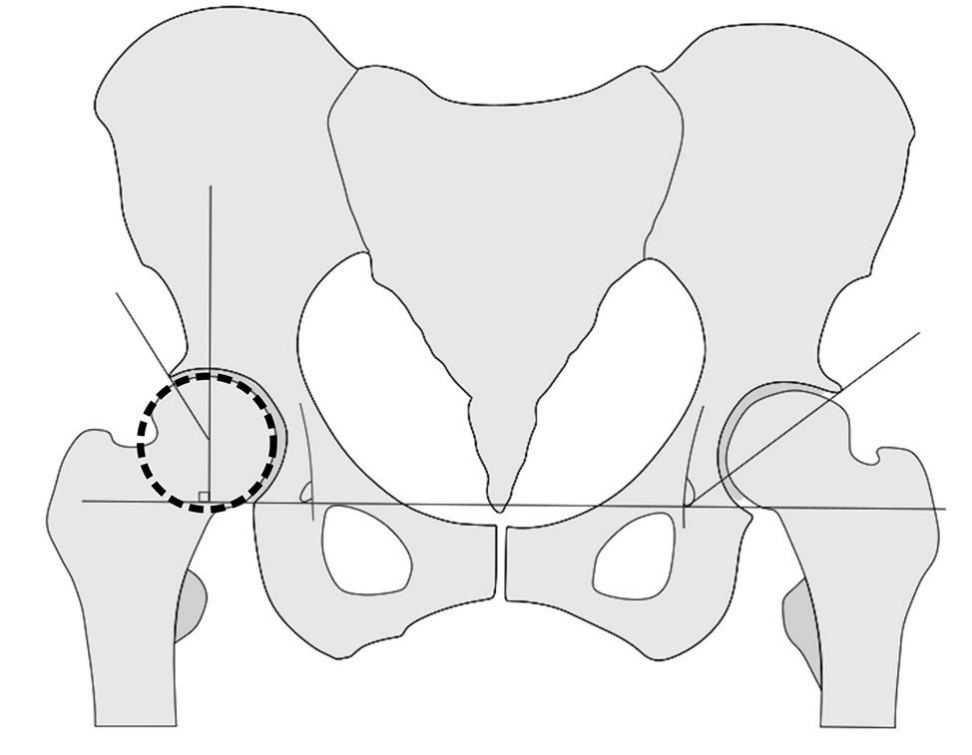
**A**: Center edge angle. **B**: Sharp’s angle

The most common radiographic measure to diagnose dysplasia is the acetabular center-edge (CE) angle (Fig. 2A) [21, 22]. The CE angle is the angle between a vertical line to the intra-teardrop line through the hip center and a line from the hip center to the boney lateral edge of the acetabulum. The CE angle measures the acetabular coverage of the femoral head. We used the original definition of the CE angle and measured the most lateral boney edge of the acetabulum. We used a matching circle on top of the femoral head to define the hip center. There is no universally accepted definition of hip dysplasia; however, a CE angle ≤ 20° is mostly considered dysplastic, whereas a CE angle between 20° − 25° is considered borderline, and a CE angle > 25° is considered normal [1, 23, 24].

**Fig. 3:**
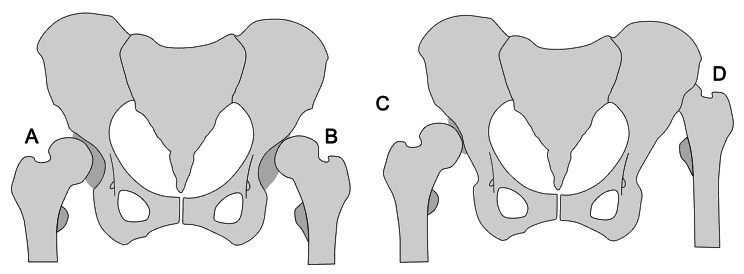
Examples of hip with different severity of hip dysplasia (**A** = Crowe 1 and Hartofilakidis 1, **B** = Crowe 2 and Hartofilakidis 1, **C** = Crowe 3 and Hartofilakidis 2, **D** = Crowe 4 and Hartofilakidis 3

To further expand the description of a dysplastic hip, the grade of subluxation can be classified. There are two such types of classification that are frequently used. The Crowe classification [25], established in 1979, uses the proportion of subluxation to divide disease severity into four classes:

- Crowe grade 1 corresponds to subluxation < 50% (Fig. 3A).
- Crowe grade 2 subluxation between 50 and < 75% (Fig. 3B).
- Crowe grade 3 subluxation between 75 and 100% (Fig. 3C).
- Crowe grade 4 total luxation (Fig. 3D).

Hartofilakidis (HA) [26] classification categorizes dysplastic hips into three classes by considering the deformation of the acetabulum in addition to the degree of subluxation:

- Dysplastic hip: The femoral head is contained within the original acetabulum despite the degree of subluxation (Fig. 3A & B).
- Low dislocation: The femoral head articulates with a false acetabulum that partially covers the true acetabulum to a varying degree (Fig. 3C).
- High dislocation: The femoral head is completely out of the true acetabulum and migrated superiorly and posteriorly to varying degrees (Fig. 3D).

The radiographs were reviewed by two reviewers (one orthopaedic surgeon, specialized in dysplastic hip surgery [ET] and one resident orthopaedic surgeon [MM]) that after a training session together, classified all images independently. All discrepant measurements were remeasured together, and the final grading was done in consensus. All hips were assessed for dysplasia by measuring the CE angle (Fig. 2A). First, using the DICOM viewer software (Horos v.3.3.6) the hip center was found for each radiograph by drawing a circle covering the femoral head. Then a horizontal line was drawn from teardrop to teardrop. The line was moved so that the end was over the circle’s center (the hip’s center). A new line was drawn from the hip’s center to the lateral edge of the acetabulum. Subsequently, the CE angle was calculated by subtracting 90 degrees from the angle between the two lines. In some hips (*N* = 10), the femoral head was too deformed to find the hip center, and for these hips, we relied on Sharp’s angle instead (Fig. 2B) [27]. A Sharp’s angle > 42° was defined as dysplastic. The CE angle is a well-established measure for hip dysplasia and was therefore chosen as the primary measure. Sharp’s angle is one of several other acetabular measures, and there is no global consensus on which is the most accurate in describing dysplasia. We chose Sharp’s angle as the secondary measure of dysplasia due to ease of measurement. Sharp’s angle is based on two easily identifiable anatomic features: the lateral acetabulum edge (the same lateral point used for the CE angle) and the teardrop. After categorizing the entire dataset into “Normal,” “Borderline,” and “Dysplastic,” we further categorized all the dysplastic hips based on both Crowe and Hartofilakidis classification. The Cohen’s Kappa [28] for interrater reliability was 0.596 for diagnosis of dysplasia (based on CE angle) before the final consensus-based grading.

### Deep learning models description

Two deep learning models were developed as follows:

- **Model 1**: To categorize all radiographs into Normal, Borderline, and Crowe 1 to 4 categories.
- **Model 2**: To categorize all radiographs into Normal, Borderline, and HA 1 to 3 categories.

Both models had a VGG16 convolutional neural network (CNN) base structure pre-trained on the ImageNet dataset [29]. VGG16 model is widely used in literature for analyzing radiographs [30]. We modified the number of neurons in the classification layer of each model according to the number of classification categories (Model 1: 6 neurons, Model 2: 5 neurons). The models were adopted for the task at hand by using a layer-wise fine-turning strategy [30]. The dataset (total 1,022 radiographs; Table 1) was divided into train, validation, and test subset containing 816, 103, and 103 radiographs respectively maintaining the same ratio in each data subset [18]. Data augmentation was used with a similar strategy as our previous work [18] to create new data by applying a series of minor translations (e.g., rotation, magnification, etc.) on the training subset to create effectively 40,000 radiographs for training. The models were trained using Adam optimizer with cross entropy loss function, for 1,000 epochs with early stoppage accuracy improvement criteria, with a batch size of 32, and an initial learning rate of 0.0001. The validation subset was used for tuning the hyper-parameters, and the test subset was used to measure the models’ performance after training. Saliency maps were implemented to indicate the importance of each pixel of a given radiograph on the models’ performance [31]. This was done for two reasons:

1. As a sanity check that the model does not use confounding data in the radiographs. One such example was Japanese letters in the Japanese radiographs that contained more dysplastic hips and subluxated hips.
2. To visualize new features that the AI could potentially find for diagnosing and categorizing hip dysplasia.

Tensorflow r1.6 with Keras backend on a workstation comprised of an Intel(R) Xeon(R) Gold 6128 processor, 64GB of DDR4 RAM, and a NVIDIA Quadro P5000 graphic card was used to implement the models.

## Results

### Data

We included pelvic radiographs from 571 patients. After exclusion, the final dataset consisted of 1,022 hip radiographs. The excluded hips either had handwritten letters and preoperative templating or a total hip arthroplasty in place. The number of images and the distribution of different classes are summarized in Table 1.

**Table 1:** The number of radiographs (hips) and classification from each contributing hospital

			Non-dysplastic			Dysplastic						
	Total		Normal	Borderline		C 1	C 2	C 3	C 4	HA 1	HA 2	HA 3
Aalborg, Denmark	95		85	8		2	0	0	0	2	0	0
Boston, USA	47		29	10		7	1	0	0	7	1	0
Emmen, Netherlands	12		8	0		4	0	0	0	4	0	0
Gothenburg, Sweden	28		15	5		8	0	0	0	8	0	0
Grapewine, USA	12		3	4		5	0	0	0	5	0	0
Hartford, USA	35		24	8		3	0	0	0	3	0	0
Hvidovre, Denmark	67		55	9		3	0	0	0	3	0	0
Kanazawa, Japan	393		74	32		122	77	71	17	187	80	20
London, UK	16		10	5		1	0	0	0	1	0	0
Madrid, Spain	20		16	2		2	0	0	0	2	0	0
New York, USA	36		27	6		3	0	0	0	3	0	0
Odense, Denmark	56		44	8		4	0	0	0	4	0	0
Oslo, Norway	50		28	9		12	0	1	0	12	1	0
Plano, USA	48		33	10		5	0	0	0	5	0	0
Stanford, USA	60		31	19		10	0	0	0	10	0	0
Uddevalla, Sweden	47		31	11		5	0	0	0	5	0	0
**Total**	**1,022**		**513**	**146**		**196**	**78**	**72**	**17**	**261**	**82**	**20**

C = Crowe, HA = Hartofilakidis

All hospitals are from data source 1 except Kanazawa (data source 2 & 3)

### Model performance

Table 2 summarizes Model 1 performance in classifying all radiographs in the test subset (103 radiographs) into Normal, Borderline, Crowe 1, Crowe 2, Crowe 3, and Crowe 4 classes. Model 1 diagnosed the normal hips with high accuracy (48 out of 51 correct classifications); however, it struggled to distinguish between Normal and Borderline hips and classified most of the Borderline hips as Normal (13 out of 15 Borderline hips were classified as Normal). On the other hand, Model 1 achieved high accuracy (92.2%) in distinguishing between dysplastic hips (Crowe 1–4) vs. Normal/Borderline hips. Considering the Crowe classification, most misclassification errors by the model were in the neighboring classes, i.e., +/- 1 Crowe classification error. Model 1 achieved an overall 68% accuracy.

Table 3 summarizes Model 2 performance in classifying all radiographs in the test subset (103 radiographs) into Normal, Borderline, HA 1, HA 2, and HA 3 classes. Model 2 also diagnosed the normal hips with high accuracy (41 out of 51 correct classifications); however, it struggled to distinguish between Normal and Borderline hips. On the other hand, Model 2 achieved high accuracy (83.3%) in distinguishing between dysplastic hips (HA 1–3) vs. Normal/ Borderline hips. Considering the HA classification, Model 2 also made +/- 1 HA classification error distinguishing between the neighboring classes. Model 2 achieved an overall 73.5% accuracy.

**Table 2:** Confusion matrix of Model 1 results, Crowe classification

	True						
	Normal	Borderline	Crowe 1	Crowe 2	Crowe 3	Crowe 4	Class precision
Predicted							
**Normal**	48	13	5	0	0	0	72.7%
**Borderline**	3	1	2	0	0	0	16.7%
**Crowe 1**	0	1	13	3	1	0	72.2%
**Crowe 2**	0	0	0	4	3	0	57.1%
**Crowe 3**	0	0	0	1	3	1	60.0%
**Crowe 4**	0	0	0	0	0	1	100.0%
**Class recall**	94.1%	6.7%	65.0%	50.0%	42.9%	50.0%	

**Table 3:** Confusion matrix of Model 2 results, Hartofilakidis (HA) classification

	True					
	Normal	Borderline	HA 1	HA 2	HA 3	Class precision
Predicted						
**Normal**	41	4	1	1	0	87.2%
**Borderline**	5	5	4	0	0	35.7%
**HA 1**	5	6	21	0	0	65.6%
**HA 2**	0	0	0	7	1	87.5%
**HA 3**	0	0	0	0	1	100.0%
**Class recall**	80.4%	33.3%	80.8%	87.5%	50.0%	

It is worth mentioning that while both Model 1 and Model 2 struggled to accurately identify the severity of dysplasia (Crowe and HA classification), they both achieved high performance in diagnosing a dysplastic hip (Normal/Borderline vs. Crowe 1–4/HA 1–3). Table 4 summarizes Model 1 performance in distinguishing between Normal/Borderline and Dysplastic hips.

**Table 4:** Confusion matrix of Model 1 results for Normal/Borderline vs. Dysplastic hip

True			
Predicted	**Normal/ Borderline**	**Dysplastic (Crowe 1–4)**	**Precision**
Normal/Borderline	65	7	90.3%
Dysplastic (Crowe 1–4)	1	30	96.8%
Recall	98.5%	81.1%	

**Fig. 4:**
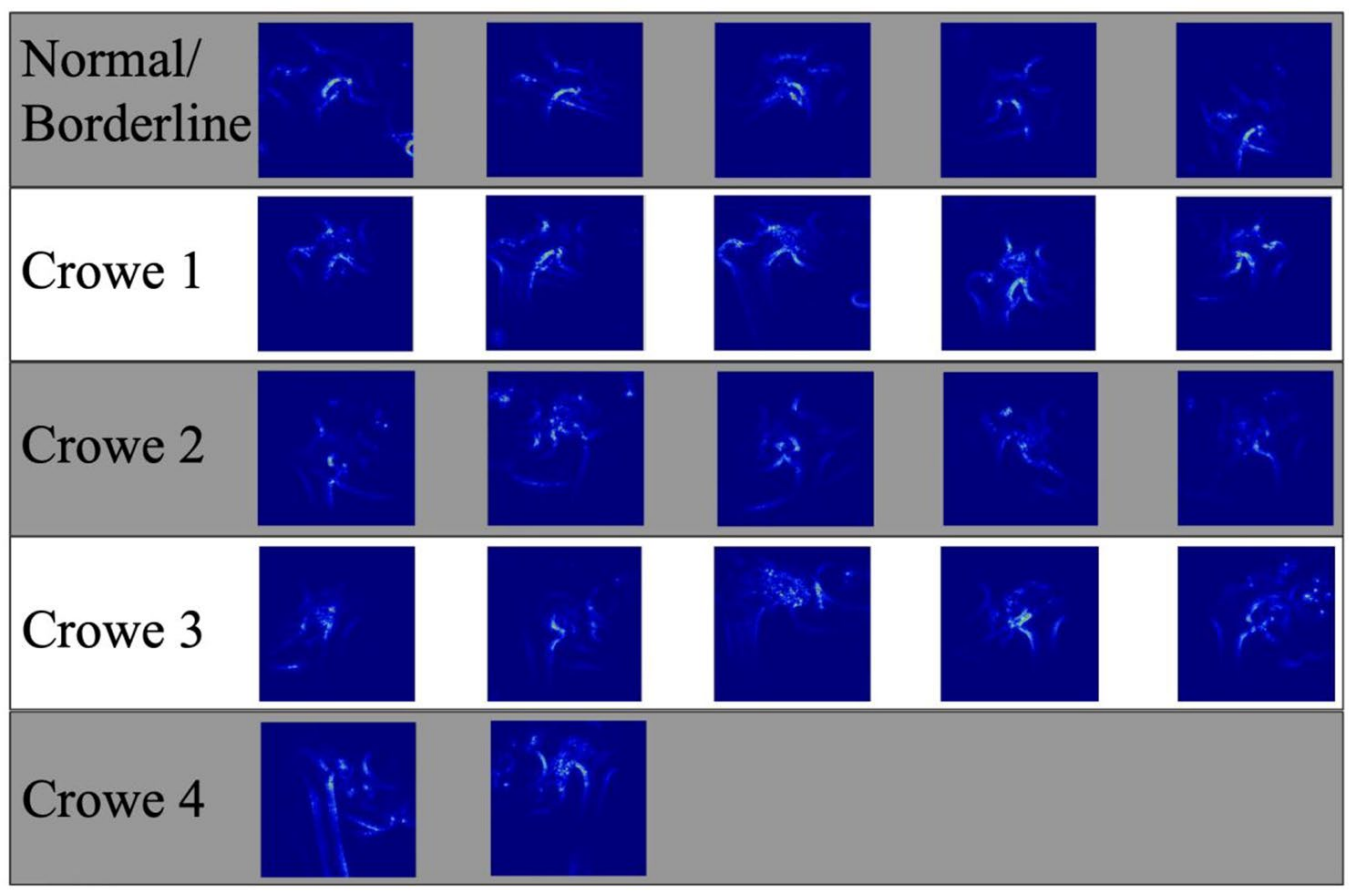
Saliency maps for Normal/Borderline and Crowe 1–4 classifications

## Discussion

### Main results

To the best of our knowledge, this is the first study to use deep learning to diagnose and classify the severity of hip dysplasia. In this study, we found that deep learning models trained on multicenter radiographs could classify hips into dysplastic or non-dysplastic with over 90% accuracy. These deep learning models were also successful in detecting the severity of hip dysplasia based on Crowe and HA classifications, where the most misclassifications were made in neighboring classes.

### Strengths and limitations

A major strength of this study is the multicenter data source across different countries and healthcare institutions. By using multicenter radiographs, the models were externally validated and thereby are suitable for global use. Furthermore, the models learned to ignore the variations in how the radiographs were performed (some centers had higher consistency than others) as well as the patients’ demographics.

Another strength is that we used two reviewers for the data labeling. This gave us a measure of the dysplasia classification quality and how hard it is to classify dysplasia consistently. After the individual reviews, all discrepancies were adjusted with consensus between the two reviewers. Although this was a time-consuming process, it resulted in more objective labels than a single reviewer. The interrater agreement between the two reviewers was moderate for diagnosing dysplasia. Both reviewers found the measurement of the CE angle to be challenging. The main challenge was defining the most lateral boney point of the acetabular edge. Only a few millimeters difference in identifying that measurement point was enough to change the CE angle and hence the classification from borderline to dysplastic/normal.

We tried to increase the understanding of the model by using saliency maps. Figure 4 shows examples of saliency maps for Normal/Borderline and Crowe 1–4 classifications. The saliency maps showed that the edge of the calcar region of the femur and its relation to the pelvic ring played an important role in the classification. This seems like a method resembling dysplasia classification by relying on Shenton’s line, which is a method that has high accuracy for determining femoral subluxation [32].

**Fig. 5:**
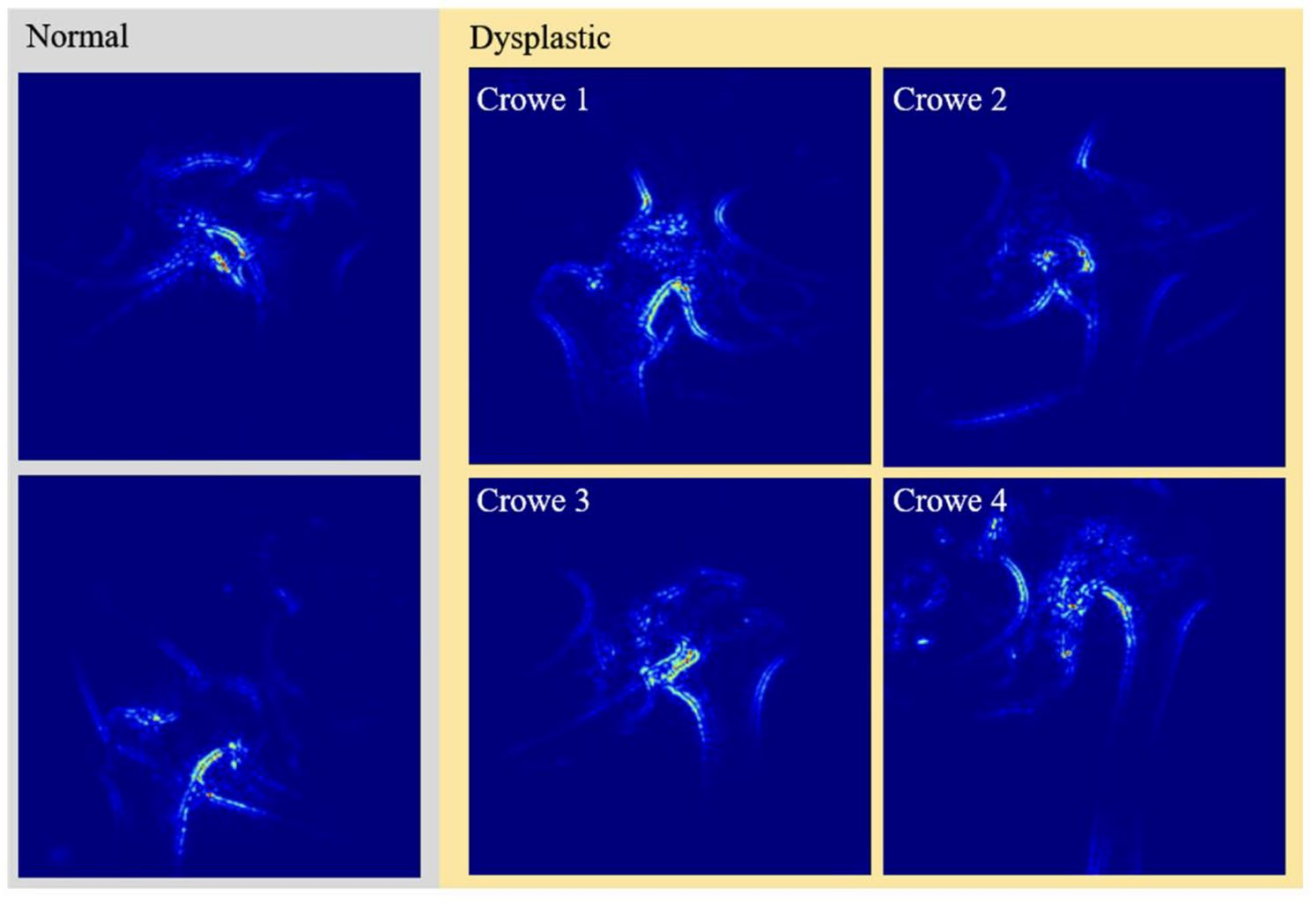
Saliency maps for example radiographs of normal and dysplastic hips. Colored regions, where red denotes a higher relative influence than blue, indicate the most influential regions on the convolutional neural network’s performance

Furthermore, the model seemed to use the inferior cortex of the femoral neck arch and its relation to the inner and outer cortices of the pelvic ring (Fig. 5). For the more dysplastic hips that also had more femoral head deformity, the model seemed to recognize a narrower arch between the neck and the deformed head (Fig. 5, Crowe class 3). Although these interpretations are highly subjective and cannot be interpreted as a “logic” being used by the model, it is encouraging to see that the model learned to focus on relevant anatomical areas without any explicit training or “rules” to follow.

This study is limited by the use of plain anteroposterior hip radiographs. The three-dimensional anatomy of the acetabulum cannot be fully captured in a two-dimensional image. For instance, the pelvic tilt and rotation can affect the CE angle and would most probably impact radiographs classification as dysplastic, borderline, or Crowe 1. There is computer software (Hip2norm) available that can compensate for the pelvic position, but to the best of our knowledge, this software has not been validated to be used for dysplastic hips [33]. Furthermore, three-dimensional modalities such as computed tomography and magnetic resonance tomography can give a more comprehensive image of the acetabular anatomy; however, plain radiographs are still the most widely used imaging modality to assess the hip joint.

There is no global consensus on the definition of hip dysplasia, and there are many different ways to measure the CE angle. A surgeon might perform several measurements and supplement them with their overall impression of the hip anatomy to make a diagnosis. This method is not as reproducible as using a single measure but could be implemented in future studies as a more clinically relevant method to diagnose dysplasia.

The dataset’s size, and more specifically the distribution of higher Crowe grade hips, is somewhat limited in this study. This is due to the lower prevalence of totally dislocated hips since these patients are usually treated before the total collapse of the hip joint. While our study yields promising outcomes in employing deep learning for hip dysplasia diagnosis, it is crucial to acknowledge that it is a pilot study and the potential for selection bias, factors that may impact the generalizability of the results. Future research with larger and diverse datasets is needed for comprehensive validation and broader applicability.

Another observation made during this study was that Crowe class 1 includes a wide range of hips from slight dysplastic with a CE angle just below 20° to more severe dysplastic hips approaching Crowe class 2. The radiograph from a slight dysplastic hip looks entirely different from a hip with, e.g., 49% subluxation. This is evident even to a person that is not used to reviewing hip radiographs. The symptoms are probably different for these two examples as well; however, they both get classified as Crowe class 1. This further explains the models’ performance in classifying the severity of hip dysplasia that they made +/- 1 Crowe or HA classification error.

Generally, the application of deep learning in the classification of adult hip dysplasia is somewhat limited. Several smaller studies, each demonstrating varying degrees of accuracy, have been conducted. For instance, Jensen et al. suggested the potential utility of their algorithm in quantifying specific landmarks of hip dysplasia, although their study was limited in size and lacked precision [34]. In another study, Archer et al. illustrated that machine learning could offer a quick and cost-saving approach for assessing certain hip dysplasia parameters, with external validation. However, their model faced challenges in identifying anatomical landmarks and had a smaller sample size compared to ours [35].

### Future implications

Hopefully, this study will encourage researchers to conduct more extensive studies with larger datasets resulting in a model with even higher performance. A similar model trained on a large dataset assessed and labeled in consensus by a global expert panel could in the future be used as a benchmark for hip dysplasia diagnosis and classification.

## Conclusion

We have developed two deep learning models to diagnose and classify hip dysplasia, archiving high performances. These models could be used by professionals who lack relevant experience and in large cohorts for automatic diagnoses and classification of hip dysplasia. Timely diagnosis of hip dysplasia and subsequent conservative treatments could potentially delay the development of OA and the requirement of more aggressive treatments.

## Data Availability

The datasets used and/or analysed during the current study available from the corresponding author on reasonable request.

## Abbreviations

AI: Artifical Intelligence
CE: Center-Edge
CNN: Convolutional Neural Network
HA: Hartofilakidis
IRB: Institutional Review Board
JPEG: Join Photographic Expert Group
OA: Osteoarthritis
THR: Total Hip Replacement
